# Pelvo-ureteric junction obstruction in the lower pole moiety of a duplex kidney with an associated intraparenchymal abscess: a case report

**DOI:** 10.1186/1752-1947-2-241

**Published:** 2008-07-24

**Authors:** James Lenton, Tze Wah

**Affiliations:** 1 Department of Radiology, St James University Hospital, Leeds LS9 7TF, UK

## Abstract

**Introduction:**

Pelvo-ureteric junction obstruction and duplex kidney are common radiological findings. However, pelvo-ureteric junction obstruction in a duplex kidney is a rare finding. We present the case of a patient who presented with septic complications secondary to this combination.

**Case presentation:**

An adult woman presented with urinary sepsis, and her initial investigation with ultrasound revealed hydronephrosis of the lower moiety of a duplex kidney. Further investigations with computed tomography and magnetic resonance imaging showed an associated intrarenal abscess and a pelvo-ureteric junction obstruction of the lower moiety of a duplex kidney.

**Conclusion:**

This patient had a rare and unreported complication of an unusual congenital urological abnormality. This case report highlights the role of multiple imaging modalities in correct diagnosis for clinical management.

## Introduction

Pelvo-ureteric junction obstruction (PUJO) is a relatively common finding during urological investigation, as is duplex kidney. PUJO is the most common cause of foetal and/or neonatal hydronephrosis [1]. Duplex kidney is the most common congenital abnormality of the urinary tract, with an incidence of around 2% [2]. However, PUJO in a duplex kidney is a rare finding. We present the case of a woman who presented with urinary sepsis secondary to an infected PUJO in the lower pole of a duplex kidney that was complicated by an intrarenal abscess.

## Case presentation

A 53-year-old British Caucasian woman presented to urologists with increasing right flank pain and pyrexia. Urine analysis was negative, and initial blood tests showed a normal white blood cell count and normal renal function. Ultrasound showed a hydronephrosis of the lower moiety of a duplex right kidney, and no cause could be identified. Unenhanced computed tomography (CT) confirmed the lower moiety hydronephrosis. A calibre change was seen in the upper ureter, but again a cause was not identified.

**Figure 1:**
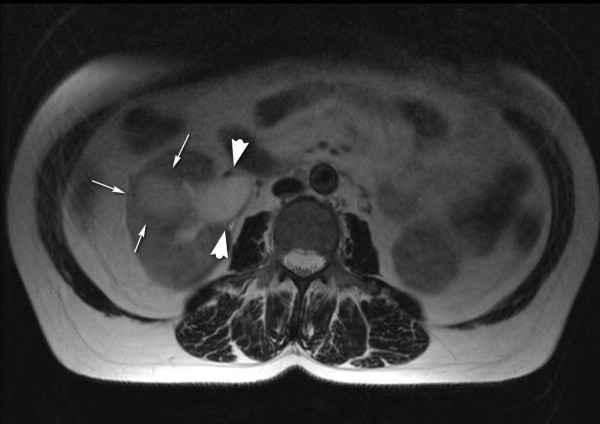
**T2-weighted axial magnetic resonance imaging scan showing a high-signal (fluid) cortical mass (arrows) with an irregular low-signal capsule**. The collecting system is dilated (arrowheads).

**Figure 2:**
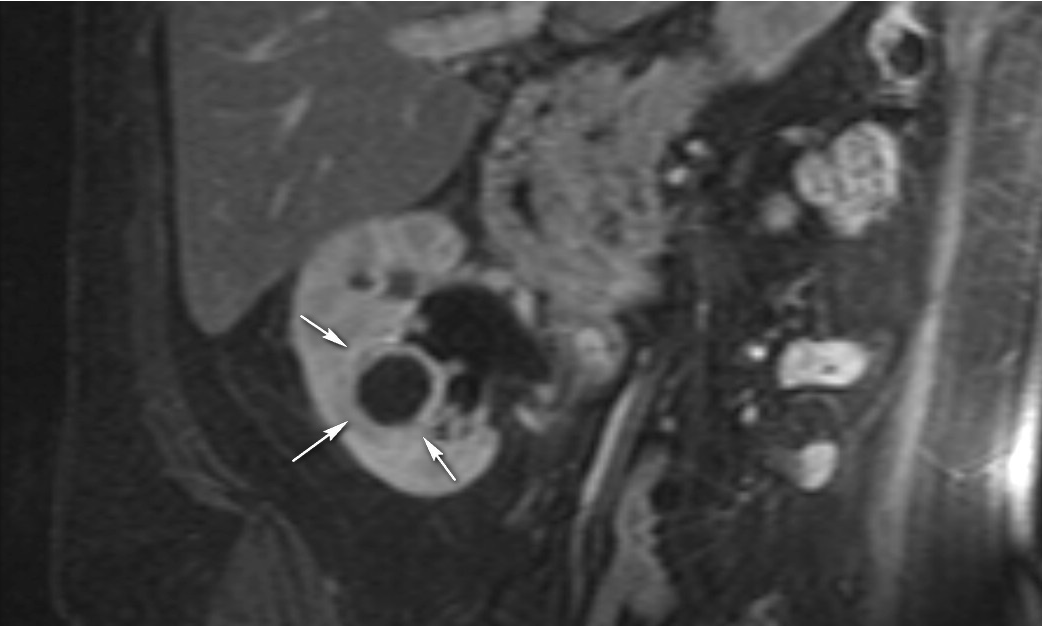
**T1-weighted coronal magnetic resonance imaging scan with fat saturation and gadolinium enhancement**. The cortical mass is seen as a low signal with an enhancing rim (arrows).

The differential diagnosis was thought to be either a small stone or tumour obstructing the upper ureter. A magnetic resonance (MR) urogram (Figures 1 and 2) showed a well-circumscribed, round lesion within the right lower pole cortex in addition to the lower moiety hydronephrosis. On T2-weighted images, this lesion had a fluid-containing centre that displayed high signal intensity with an ill-defined lower-signal-intensity capsule. On fat-suppressed post-gadolinium T1-weighted sequences, this lesion displayed a low-signal-intensity centre with an avidly enhancing capsular rim. On the MR appearances, the differential diagnosis was either a tumour or an abscess. Given that the patient was septic, this cystic lesion with an enhancing capsule was likely to be an abscess rather than a tumour. Again no extrinsic cause for the obstruction was demonstrated.

The patient underwent ultrasound-guided nephrostomy and a guided aspiration of the fluid collection. This yielded thick pus from the collection and clear urine via the nephrostomy. A subsequent microbiological analysis of the pus and urine showed no growth. However, she had received 3 days of cefuroxime. These two procedures led to the resolution of the pain and pyrexia.

**Figure 3:**
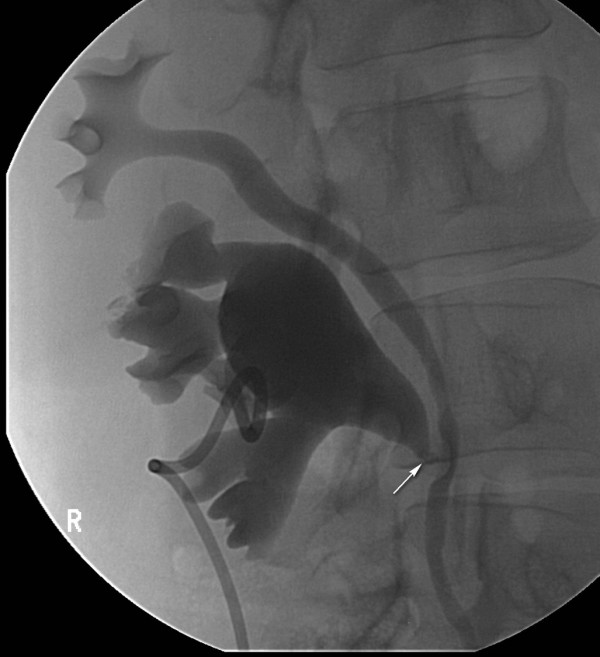
**The nephrostogram shows the dilated lower moiety with an abrupt transition to non-dilated ureter at the pelvo-ureteric junction (arrow) consistent with pelvo-ureteric junction obstruction of the lower moiety**. The upper moiety is non-dilated.

A nephrostogram (Figure 3) performed several days later showed an incomplete duplex with the ureter joining at the pelvo-ureteric junction (PUJ). The lower moiety was moderately hydronephrotic and had a PUJO configuration. The upper moiety was non-hydronephrotic. The lower single ureter had a normal appearance with no evidence of vesico-ureteric obstruction. MAG 3 renography confirmed no significant obstruction. In view of this, the PUJO was believed to likely be mild or intermittent. The patient remained asymptomatic 7 months later, and no further treatment is planned.

## Discussion

Duplex systems may be complete or more commonly incomplete. The complete duplications are usually from a second ureteral bud, and the incomplete duplications are a result of the splitting of the ureteral bud [1].

In a completely duplicated system, the upper moiety ureter inserts inferior and medial to the correctly sited ureter draining the lower moiety. In this situation, the upper moiety is more susceptible to obstruction. This may be due to a ureterocele, to ectopic insertion of the ureter, or to an aberrant artery. The lower pole moiety is prone to reflux because of an abnormal but correctly sited vesico-ureteral junction. The angle the ureter takes through the bladder wall is more acute. This arrangement can give rise to the 'drooping lily' sign seen on intravenous urography; excretion is only in the lower moiety, which is displaced and rotated by the dilated non-functioning or poorly functioning upper moiety [1].

In retrospect, in our patient the abnormally thickened upper ureter on unenhanced CT must have reflected the swollen and convoluted junction of the two moieties.

The causes of a hydronephrotic lower moiety in a duplex system include reflux, vesico-ureteral junction obstruction secondary to a ureterocele of the upper moiety insertion, PUJO, calculi, intrinsic tumours and external compression. Upper moiety obstruction is seen secondary to ectopic ureterocele and ureteral insertion in the case of complete duplex kidney, or due to an aberrant artery. PUJO in a duplex system is rare but has been reported in both the upper and the lower moiety in completely and incompletely duplicated systems [3].

It has been suggested that reflux may play a role in the development of PUJO in duplex systems, with peri-ureteric inflammatory change causing a fixation of the PUJ, leading to persistent PUJO [4]. Vesico-ureteral reflux to the lower moiety is usually associated with complete duplex systems, whereas uretero-ureteral reflux may occur in partial duplex systems [1].

Sometimes a diagnosis may be made in adulthood, for example when the patient presents acutely with pyonephrosis. In an acute presentation with pyrexia, percutaneous nephrostomy is the treatment of choice [2]. Elective treatment depends on the individual anatomy and the level of function of the lower moiety. If the lower moiety is non-functioning, heminephrectomy is an option, particularly if the patient is symptomatic. If there is reasonable function, then renal preservation needs to be considered. If the ureter is short, then a lower-to-upper-pole pyeloureterostomy may be considered. If the ureter is longer, a more conventional pyeloplasty is appropriate [5].

## Conclusion

We have discussed a rare and, to the best of the authors' knowledge, previously unreported complication of an unusual congenital abnormality. This case report demonstrates the important role of multiple imaging modalities in providing the information needed to arrive at the eventual accurate diagnosis and in order to provide the patient with optimal clinical management.

## Abbreviations

CT: computed tomography; MR: magnetic resonance; PUJ: pelvo-ureteric junction; PUJO: pelvo-ureteric junction obstruction.

## Competing interests

The authors declare that they have no competing interests.

## Authors' contributions

JL gathered the information, reviewed the literature and drafted the manuscript. TW critically appraised the manuscript. All authors have read and approved the final manuscript.

## Consent

Written informed consent was obtained from the patient for publication of this case report and any accompanying images. A copy of the written consent is available for review by the Editor-in-Chief of this journal.
